# Assessment of cleaning and disinfection of Intensive Care Unit beds: quality management with Lean

**DOI:** 10.1590/0034-7167-2025-0316

**Published:** 2026-10-19

**Authors:** Joana de Oliveira Pantoja Freire, Christiany Moçali Gonzalez, Augusto da Cunha Reis, Robisom Damasceno Calado, Marluci Andrade Conceição Stipp, Graciele Oroski Paes

**Affiliations:** I Universidade Federal do Rio de Janeiro. Rio de Janeiro, RJ, Brazil; II Instituto Federal de Educação Tecnológica Celso Suckow da Fonseca. Rio de Janeiro, RJ, Brazil; III Universidade Federal Fluminense. Rio das Ostras, RJ, Brazil

**Keywords:** Disinfection, Housekeeping, Hospital, Quality Indicators, Health Care, Total Quality Management, Intensive Care Units., Desinfección, Servicio de Limpieza en Hospital, Indicadores de Calidad de la Atención de Salud, Gestión de la Calidad Total, Unidades de Cuidados Intensivos.

## Abstract

**Objective:**

to analyze, using Lean Healthcare tools, the terminal hygiene process in Intensive Care Unit beds and to propose improvements for its standardization.

**Method:**

a cross-sectional study was conducted at a university hospital in Rio de Janeiro between September 2023 and February 2024, observing ten terminal hygiene processes performed by five professionals. VSM, CRT Diagram, GUT Matrix, Prioritization Matrix, and 5W2H tools were used. Analysis considered scope, technical adherence, execution time, surface completeness, and the presence of fluorescent residues.

**Results:**

failure to use protective equipment and disinfectant was identified in 100% of procedures, along with a lack of technical standards and movement patterns. Only 70% of processes were completed, and 33% of surfaces were free of residue.

**Conclusion:**

despite institutional guidelines, hygiene was not standardized. Lean tools allowed for the identification of root causes and the development of improvement strategies focused on the real existing problems.

## RESUMEN

**Objetivo:**

analizar, mediante herramientas Lean Healthcare, el proceso de higiene terminal en camas de unidades de cuidados intensivos y proponer mejoras para su estandarización.

**Método:**

estudio transversal realizado en un hospital universitario de Río de Janeiro entre septiembre de 2023 y febrero de 2024, observando diez procesos de higiene terminal realizados por cinco profesionales. Se utilizaron las herramientas VSM, Diagrama ARA, Matriz GUT, Matriz de Priorización y 5W2H. El análisis consideró el alcance, el cumplimiento técnico, el tiempo de ejecución, la integridad de la superficie y la presencia de residuos fluorescentes.

**Resultados:**

se detectaron fallos en el uso de equipos de protección y desinfectante en el 100% de los procedimientos, además de la falta de estándares técnicos y patrones de movimiento. Solo se completó el 70% de los procesos y el 33% de las superficies quedaron libres de residuos.

**Conclusión:**

a pesar de las directrices institucionales, la higiene no estaba estandarizada. Las herramientas Lean permitieron identificar las causas raíz y desarrollar estrategias de mejora centradas en los problemas reales existentes.

## INTRODUCTION

Healthcare-associated infections (HAIs) are a significant public health issue. They are recognized by the World Health Organization for their association with high morbidity and mortality rates^(1,2)^. Although some HAIs are unavoidable, many can be prevented by adopting proper practices such as hand hygiene and standard precautions^(2,3)^.

Direct or indirect cross-transmission is a key factor in the acquisition of these infections, with environmental contamination by microorganisms from colonized patients having a strong influence^(3)^. In this context, Intensive Care Units (ICUs) stand out, as they are characterized by greater clinical severity, frequent use of invasive devices, and consequently, greater vulnerability to colonization by pathogens present in the environment^(2)^.

Studies show that contaminated surfaces can significantly increase the risk of infection, as hospital-acquired microorganisms remain viable for extended periods^(3,4)^. Proper hygiene of beds, including mattresses, bed rails, and headboards, is essential for HAI prevention, especially because frequently touched surfaces near the patient act as reservoirs and vectors of multidrug-resistant microorganisms^(2,3,5)^.

Cleaning, which precedes disinfection, removes visible dirt and provides comfort to patients, while disinfection aims to eliminate microorganisms, ensuring greater safety^(3)^. When performed systematically and validated, hospital hygiene contributes significantly to patient safety^(2,6)^.

Despite this, failure in hygiene processes is common, such as low adherence to technique, lack of monitoring, and waste. Studies point to worrying rates of non-compliance in ICUs, with inadequate cleaning in critical areas^(7,8)^. To address these shortcomings, continuous improvement in the quality of these processes is necessary, involving nursing as a key player in hygiene management^(9)^.

Terminal hygiene (TH) assessment in ICUs has become a focus of studies aimed at safety of care. A promising approach in this scenario is Lean Healthcare, which applies principles of lean thinking to reduce waste and optimize processes^(6,10)^.

Originating in the automotive industry, Lean is a philosophy based on continuous improvement, standardization, and delivering value at the lowest possible cost^(11)^. In healthcare, Lean Healthcare has proven effective in improving quality of care, promoting better outcomes through the rational use of resources^(10,11)^. Hospitals in Brazil and abroad have made significant progress with this approach, including greater operational efficiency, reduced errors and waiting times, as well as a positive impact on patient experience^(12-14)^.

While still in its early stages in the field of nursing, the application of Lean requires further studies on its impacts on care management and clinical outcomes. The COVID-19 pandemic accentuated the need for solutions that combine efficiency and quality, boosting the use of management tools such as Lean^(11)^.

The relationship between environmental contamination and acquisition of HAIs is widely recognized, especially through indirect transmission mediated by frequently touched surfaces^(4)^. In this context, bed TH constitutes a strategic component in infection prevention, but it still presents technical variability and opportunities for improving care quality. The use of Lean Healthcare in this scenario makes it possible to analyze the bed as a micro-circuit of care, identifying waste, mapping operational flows, and measuring results with objective parameters, such as the fluorescent marker (FM)^(10-12)^. This approach provides greater diagnostic accuracy to the process and supports standardized and reproducible interventions, reinforcing the originality and relevance of this study for patient safety and for the scientific and managerial advancement of Brazilian nursing.

Given that frequently touched hospital surfaces are involved in the transmission of microorganisms, it becomes imperative to adopt systematic interventions to promote the continuous improvement of cleaning and disinfection (CD), ensuring safe and effective practices.

## OBJECTIVE

To analyze, using Lean Healthcare tools, the TH process in ICU beds and propose improvements for its standardization.

## METHODS

### Ethical aspects

The research was approved by a Research Ethics Committee, following the principles of Resolution 466/2012, as well as the precepts of autonomy, non-maleficence, beneficence, justice, and equity. All participants signed the Informed Consent Form.

### Study design

This is a cross-sectional observational study with a quantitative and descriptive approach, conducted according to the STrengthening the Reporting of OBservational studies in Epidemiology checklist. The Lean Healthcare principles were applied, along with tools from the Theory of Constraints (TOC) (Current Reality Tree - CRT) and Total Quality Management (GUT Matrix, 5W2H, Prioritization Matrix, and Spaghetti Diagram), to map and analyze the bed occupancy rate process in an ICU.

The literature recommends integrating these tools as an effective practice in managing and improving healthcare processes as it promotes robust diagnoses and sustained interventions^(15)^. Lean Healthcare functions as a flexible methodological framework, allowing for combination with approaches such as Six Sigma, TOC, and Total Quality Management^(11)^.

Additionally, FM was used as an objective instrument to assess the technical conformity of the cleaning process, serving as an indicator of operational quality. This technique measured whether the critical contact points were correctly cleaned, reinforcing the findings of Lean tools^(5,7)^.

The tools were organized into three phases, adapted to the continuous improvement cycle of Lean Healthcare, such as process mapping, root cause analysis, and proposing sustainable solutions.

### Phase I - Mapping, measuring, and identifying process flaws

The tools used were Value Stream Mapping (VSM), Spaghetti Diagram, and FM. VSM allowed visualization of the TH flow, identifying sequence failures, rework, idle time, and technical adherence. The Spaghetti Diagram, adapted with a numerical sequence in a floor plan, represented professionals’ movements, highlighting wasted time and ergonomic issues. FM, applied blindly by the researcher, allowed verification of the effective cleaning of the previously selected surfaces^(16,17)^.

### Phase II - Root cause analysis of problems

The tools used were CRT and GUT Matrix. TOC’s CRT allowed for the logical mapping of undesirable effects and their root causes. The GUT Matrix helped prioritize critical failures according to severity, urgency, and trend, guiding improvement actions^(18,19)^.

### Phase III - Proposal for improvements

The tools used were the 5W2H Action Plan and the Impact x Effort Matrix. Based on the findings, an action plan was structured using 5W2H, clearly organizing what, how, why, and by whom the actions would be executed. The Impact x Effort Matrix helped prioritize the most feasible tasks with the highest operational return^(19)^.

### Study period and setting

The study was conducted between September 2023 and February 2024 in the ICU of a public university hospital in Rio de Janeiro. The ICU has nine beds, with a mean of 20 admissions per month and a mean length of stay of 15 days per patient^(20)^.

### Population, sample, and inclusion and exclusion criteria

The sample was a census taken during the daytime period, comprising the five professionals responsible for cleaning beds in the ICU (four on 12x36 hour shifts and one day shift worker), corresponding to 100% of the staff working during the shift in which most terminal hygiene occurs. Each professional performed two procedures, totaling ten observations.

There were also four professionals on the night shift (12x36); however, this shift was not included in data collection because it had a lower incidence of discharges and, consequently, a lower number of TH during the study period.

ICU cleaning professionals, with a minimum of three months of experience in the sector and prior completed institutional training for cleaning in a Critical Care Unit, were included. Data collection exclusively covered the daytime period, when there is a higher volume of human hemorrhagic fluid. Professionals on vacation or leave during the collection period, as well as those whose observations were interrupted by events that prevented full monitoring of the procedure, were excluded.

Bed selection followed a recommendation from the Centers for Disease Control and Prevention, which advises assessing at least 25% of beds in Critical Care Units^(21)^. Criteria were defined for selecting the surfaces to be assessed with FM based on in situ observations and scientific literature^(1,3,4)^.

The surfaces analyzed included the infusion pump (front and side), the pump stand, the enteral pump, the dressing cart, the mattress, the side rails (inner and outer), the bed controls, the monitor, the gas regulator, the feeding table, the switch, the handles, the bedside table, the monitor buttons, the alcohol dispenser, and the privacy screen.

### Data collection

The study was conducted in three phases: phase 1 - direct observation of TH, with completion of VSM (times, interruptions, conformities and non-conformities according to institutional protocol). FM (Glo Germ^®^) was applied to the surfaces before sanitization, always by the same researcher, ensuring operational confidentiality. The Spaghetti Diagram was developed based on the sequence of cleaning actions on the ICU floor plan, mapping movements and techniques employed^(22)^; phase 2 - classification and organization of problems into a logical structure using the CRT method, followed by the application of the GUT Matrix for prioritization; Phase 3 - Structuring the action plan with 5W2H and prioritization using the Impact x Effort Matrix, according to the feasibility and impact of the proposed actions.

### Data analysis

The main variables were dependent (quality of the cleaning process - scope and technical adherence according to the Standard Operating Procedure (SOP)) and independent (execution time, interruptions, use of personal protective equipment (PPE), order and technique of cleaning).

Descriptive statistics (frequencies, percentages, and means) were used to analyze process performance. VSM and Spaghetti Diagram tools were constructed using Miro^®^ and CorelDRAW^®^ platforms. Fluorescence assessment was performed using UV LED light, classifying the residue intensity into four levels (Figure 1). The action plan was modeled based on the 5W2H and the Impact x Effort Matrix^(7,12,22)^.

**Figure 1:**
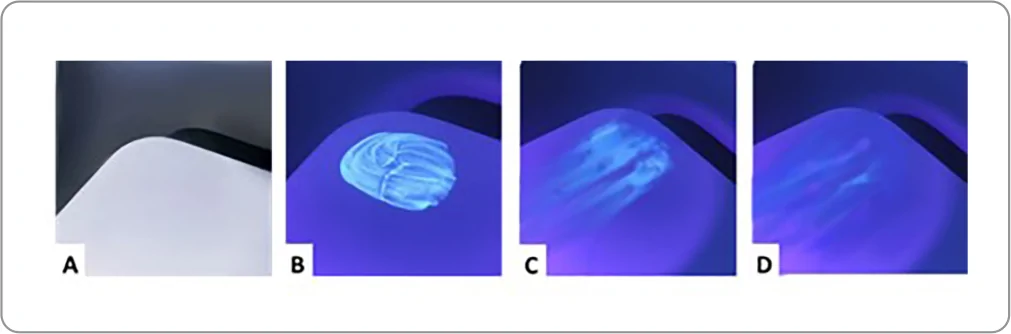
Parameters for analyzing fluorescent residue on surfaces. Rio de Janeiro, RJ, Brazil, 2024

*Legend: A = surface in normal light; B = intense residue; C = moderate residue; D = light residue.*

## RESULTS

### Phase I - Identifying and measuring the problems

The VSM tool enabled the mapping of the current state of the bed TH process, the identification of the main non-conformities that directly interfere with its cadence, as well as the verification of the mean time of 3 hours and 10 minutes (53 minutes to 4 hours and 19 minutes) for the bed to be released for a new admission (Figure 2).

**Figure 2:**
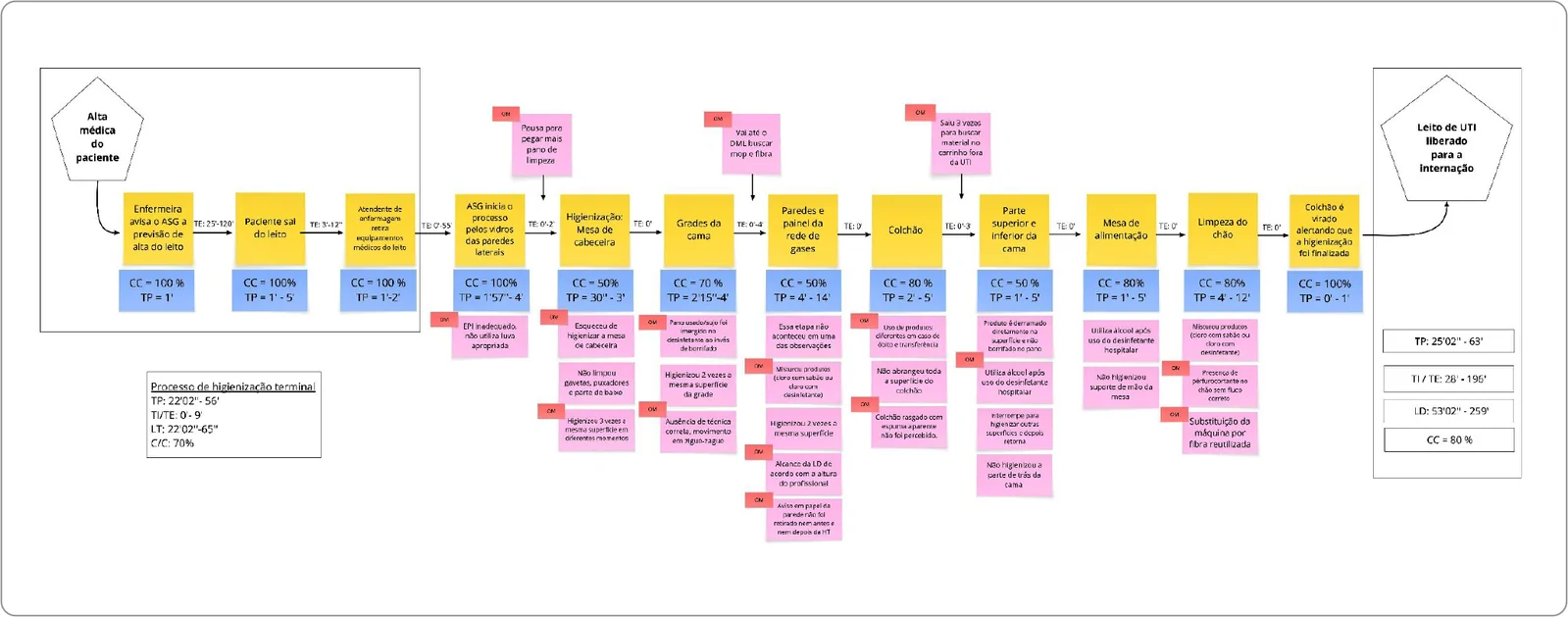
Mapping the current state of terminal hygiene in the Intensive Care Unit. Rio de Janeiro, RJ, Brazil, 2024

*Legend: PT = process time; WT = waiting time; IT = interruption time; LT = lead time; CC = complete/correct; OI = opportunity for improvement.*

The factors that impacted the start time and quality of bed cleaning were the availability of beds in medical and surgical wards, the urgency demanded by a physician for the admission of new patients, and cleaning staff workload.

When examining only the sanitization process, the minimum and maximum times spent from start to finish were significant, ranging from 22 to 56 minutes. Waiting time and interruptions at each stage occurred mainly to retrieve cleaning materials.

In all observations, at least one surface was not included in the cleaning procedure (bedside table, drawers, drawer handles, high wall, headboard, feeding table support, and part of the mattress). On the other hand, the feeding table, bed rails, wall areas, and bedside table received mechanical friction in a quantity exceeding that recommended in the institutional SOP, since the process performed only once, completely and correctly, would be effective.

The terminal hygiene process for the floor was carried out with a solution composed of water, soap, hypochlorite, and a disinfectant based on quaternary ammonium compounds and biguanide. Seventy percent alcohol was also frequently used after applying the hospital disinfectant, constituting a second disinfection of the fixed surfaces.

Inadequacies were also identified in the use of PPE, in the application of the cleaning technique (characterized by movements that did not cover all surfaces), in the practice of using sanitizers (through the immersion of soiled cleaning wipes in disinfectant, with a return flow to clean areas), and in the establishment of different hygiene routines for discharge and death.

The presence of sharps on surfaces without immediate disposal and without notification to the multidisciplinary staff, as well as the lack of communication regarding loss of quality/integrity or damage to equipment and/or materials, was identified as a need for process improvement.

From the observations that led to the construction of VSM, end-to-end compliance was not achieved, resulting in a percentage of 70% for the complete and correct indicator in the TH process and 80% in the ICU admission and discharge macro-process.

The flaws in the application of the hygiene technique in the patient unit, identified in VSM, were illustrated in the adapted Spaghetti Diagram, shown in Figure 3. The main inadequacies in the process were areas not covered (bedside table and drawers) and various movements, such as back-and-forth, zigzag, and circular motions, noting that no movement was similar to another in terms of order and direction. The staff’s rework in cleaning the same surface two to three times and the lack of flow and standardization of movement were other aspects observed.

**Figure 3:**
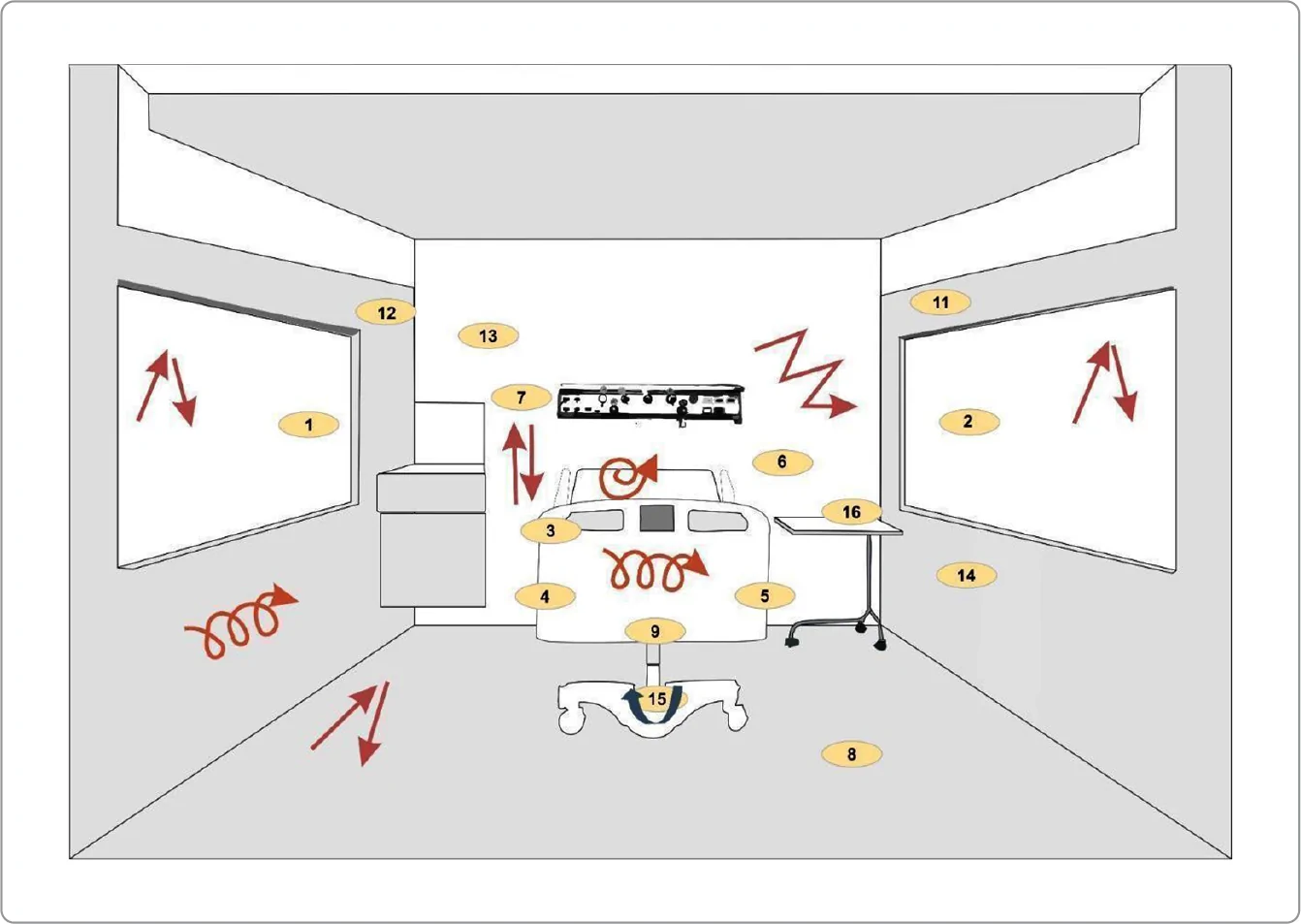
Diagram of the order and flow of terminal hygiene in the Intensive Care Unit. Rio de Janeiro, RJ, Brazil, 2024

During one of the inspections, there was an informational paper stuck to the bed and another to the wall with adhesive tape, which were not removed before the cleaning process took place. There was a lack of cleaning of the walls and the gas rail due to the height of the professional performing the cleaning, who also did not check/retrieve the cleaning support cable.

The use of FM generated 221 assessments of 20 types of surfaces that make up the patient’s environment, and not the care environment as a whole.

The application of the FM test supported the assessment of the quality of the CD technique, which made it possible to identify failures and absences of CD, including surfaces that were visually completely and correctly sanitized in the VSM. The proper execution of the CD technique, represented by the complete removal of residue, accounted for approximately one-third of all surfaces assessed.

The only areas that achieved 50% or more cleanliness compliance, as expressed by the absence of residue, were infusion pump support (75%), dressing cart surface (54%), and outer bed rail, front infusion pump, cart support, and feeding table (50%).

The privacy screen, alcohol dispensers, and enteral pump obtained results considered non-compliant in all observed treatment tests, as they presented 100% residue. The privacy screen, bedside alcohol dispenser, gas network panel, enteral pump, switches, and side infusion pump showed intense residue above 50% in the marker assessment. Of the 221 areas assessed, 33% of the highly touched surfaces did not suffer any type of CD, demonstrating that there was no local mechanical friction.

### Phase II - Problem analysis

To understand why the TH process did not occur in a standardized way, several connections between the identified problems were made through various “whys”. Some similar or repeated problems were removed, while others, subjective and identified from the statements of the professionals executing the process, were included, as shown in Figure 4.

**Figure 4:**
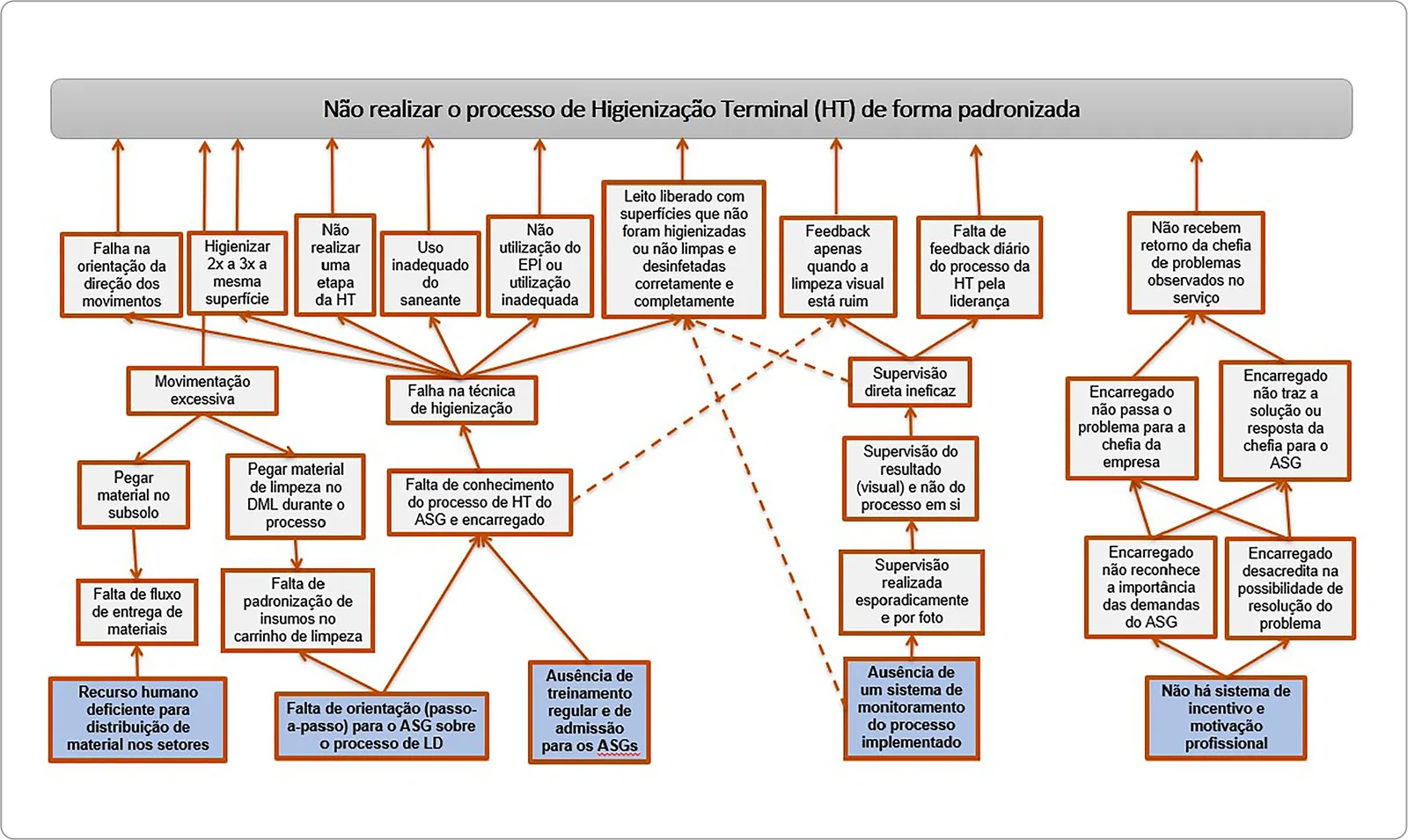
Current Reality Tree of problems encountered in the terminal hygiene process at the bedside in the Intensive Care Unit. Rio de Janeiro, RJ, Brazil, 2024

After analyzing the problems and their causal relationships, five root causes were identified for the non-implementation of TH according to SOP: insufficient human resources for material distribution in the sectors; lack of guidance (step-by-step) for hospital cleaners (HCs) on the CD process; absence of regular training and admission for HCs; absence of an implemented process monitoring system; and absence of a professional incentive and motivation system.

According to Table 1, the root causes were scored on the GUT Matrix to define which problems are priorities and deserve more urgent resolution.

**Table 1:** GUT Matrix of root causes of non-standardized terminal hygiene. Rio de Janeiro, RJ, Brazil, 2024

Problem	Gravity	Urgency	Trend	G x U x T	Classification
Lack of step-by-step guidance for hospital cleaners regarding the cleaning and disinfection process	5	5	5	125	**1^st^**
Lack regular and onboard training of hygiene professionals	5	5	5	125	**1^st^**
Lack of a process monitoring system	5	4	5	100	**2^nd^**
Lack of a checklist for authorizing the start and end of the cleaning process	5	5	3	75	**3^rd^**
Inefficient communication channel between the company and the healthcare department	3	4	4	48	4^th^
Deficient Human Resources department for material distribution across departments	3	2	2	12	5^th^

Due to the lack of a routine for initial training and continuing education of professionals performing TH, a lack of knowledge in following the step-by-step process was observed. Furthermore, in case of doubt, there is no readily available instructional material explaining the correct technique, movement, and order of CD.

### Phase III - Proposal for improvement

The action plan based on the 5W2H tool addressed the implementation of eight improvement actions to solve the main problems related to the hospital cleaning process, as defined in the GUT Matrix. The solution actions include:

1. Facilitate the staff’s understanding and access to SOP by creating an illustrated guide that clearly describes the step-by-step cleaning process (1^st^ problem);
2. Train the cleaning staff in a dynamic and engaging way, using materials such as poster boards and games, in order to reinforce learning of SOP (1^st^ problem);
3. Implement initial training for new employees, identifying and training instructors who will be responsible for welcoming and training new employees (1^st^ problem);
4. Introduce the instructors to the staff and acknowledge their contribution through institutional marketing and the awarding of certificates, highlighting professional recognition (1^st^ problem);
5. To train supervisors on the standardized cleaning and leadership process through specific training that utilizes SOP and leadership tools (1^st^ problem);
6. Implement a system for frequent monitoring of the TH process using indicators such as FM and provide continuous feedback to the staff (2^nd^ problem);
7. Create a “D-Day” for hospital cleaning professionals in conjunction with marketing, celebrating their results and promoting recognition for the work done (2^nd^ problem);
8. Create and implement a bed release checklist to ensure that all cleaning stages are verified before release for new admissions and create a visual alert for cleaned and uncleaned beds (3^rd^ problem).

After brainstorming possible solutions to the problems, defining what to do, why and how to do it, where it will be done, the cost of each action, the intervention period and, finally, who will be responsible for the improvement action, the activities were prioritized according to the effort and impact of each task.

After analyzing the Effort (horizontal axis) x Impact (vertical axis) Matrix, the actions that were classified as having the least effort and impact were: developing an annual training schedule; creating a feedback system for the TH process for HCs; and creating and implementing a bed release checklist. The second priority action, corresponding to the quadrant requiring the most effort and impact, included: implementing structured onboarding and continuing training; creating an illustrated booklet with step-by-step instructions for hygiene techniques; creating and implementing a hygiene assessment indicator; and training trainers and knowledge multipliers for the hygiene process. Finally, the action requiring the most effort but with the least immediate impact would be the creation of a HC Day as an institutional event.

## DISCUSSION

This study, innovative in the national context, investigated the shortcomings and opportunities for improvement in the ICU bed TH process from a Lean Healthcare perspective^(10-13)^. The application of VSM and other tools enabled a detailed visualization of the process, revealing technical non-conformities, interruptions, rework, and waste that compromise efficiency and safety^(11,16)^.

Among the main findings, rework stands out as a recurring waste: surfaces were cleaned multiple times without any gain in effectiveness, consuming time and resources unnecessarily. According to Lean principles, this practice reflects the categories of unnecessary movement waste, wasted resources, and inadequate processes^(14,16)^.

The observed technical variation, combined with the time spent and the morphology of surfaces, also affected the quality of the cleaning. Studies indicate that smooth surfaces tend to be cleaned more effectively than irregular surfaces, which, although more challenging, require greater attention and friction, something that was not always observed^(4,5)^.

Significant flaws were identified regarding the use and handling of sanitizing agents, such as improper dilutions, mixing of products, and immersion of contaminated cloths in the disinfectant solution, which compromised the process. Failure to adopt the two-bucket technique and the lack of knowledge of recommended practices contributed to the perpetuation of these non-conformities, with the risk of cross-contamination, as demonstrated in previous studies, and dilution of disinfectants, which can reduce their effect or even inactivate the active ingredient^(23)^.

The simultaneous use of 70% alcohol and hospital disinfectant, both with similar purposes, constituted rework. These findings reveal not only waste but also gaps in technical skills, highlighting the need for educational interventions and operational standardization, as advocated by the Kaizen principle (continuous improvement)^(11,12)^.

The mean observed hospital stay time (3 hours and 10 minutes), considered high, directly impacts the availability of beds for new patients. An international study demonstrated that the application of VSM and Kaizen in a hospital setting can significantly reduce this time, resulting in gains in efficiency and patient satisfaction^(10,16)^.

Despite the established routine for the disposal of sharps, occasional failures were observed, such as failure to dispose of sharps immediately and lack of internal notification. This weakness exposes professionals to occupational risks, indicating a need for reinforcement of protocols and training^(3,5)^.

Material integrity also interfered with the effectiveness of the cleaning. Damaged surfaces, such as torn mattresses, make adequate disinfection impossible and promote biofilm formation. The adoption of visual inspections, audits, and impermeability tests is fundamental for patient safety^(4,23)^.

Direct observation revealed the absence of a technical standard in the TH stages. The adapted Spaghetti Diagram highlighted variations in the sequence of actions and movements, reinforcing the need for standardization. The use of FM complemented this analysis, revealing surfaces that appeared clean but, under UV light, showed intense residue, suggesting flaws in the cleaning technique^(8,16,21)^.

When the missing residues (indicators of complete and correct cleaning) were isolated, it was found that only about 33% of the surfaces met the criteria. This means that, for every ten beds available, only three would be adequately sanitized to receive a new patient^(5,6)^.

Studies demonstrate that the use of FM combined with immediate feedback can improve adherence to correct cleaning practices. In the present study, a lack of systematic feedback on professionals’ performance was observed, with feedback, when present, being predominantly negative. Positive and educational feedback has the potential to act as an incentive and to value the cleaning staff’s work^(5,21,24)^.

Analysis using CRT revealed that the main causes of failures were the lack of formal guidance on the step-by-step process of TH, the absence of systematic training, and the lack of reference materials for professionals. This reinforces the importance of developing structured training actions, promoting critical thinking, and implementing a culture of continuous improvement^(11,17)^.

The development of action plans based on the 5W2H method and the prioritization of tasks using the Impact x Effort Matrix allowed for the outlining of viable and high-impact proposals. Among these, the creation of a checklist for the start and end of TH stands out, aligned with SOP and validated by VSM observations, aiming to ensure process safety and facilitate its execution^(12)^.

Lean thinking offers a strategic approach to reviewing and improving healthcare processes. By eliminating waste and promoting standardization, it contributes to patient safety, operational efficiency, and the sustainability of services. Although its application is still limited in the area of hospital hygiene, the results of this study reinforce its transformative potential^(10,13)^.

### Study limitations

Despite its methodological consistency, this study has limitations that must be acknowledged. Data collection occurred in a single ICU, which restricts the extrapolation of findings to other settings with different care dynamics, physical structures, and organizational cultures. The sample, although small, was a census during the daytime period, encompassing all professionals responsible for bed cleaning, which justifies the sample size and mitigates selection biases. However, the exclusion of the night shift may have limited the behavioral variability analyzed. Another limitation refers to the absence of microbiological assessment of surfaces, which could complement and deepen the findings obtained with microbiological testing and direct observation. Nevertheless, the instruments used demonstrated adequate sensitivity to identify technical flaws and guide improvements in the cleaning process, suggesting the potential for replicating the analysis model in other units and institutions, through adaptation to the local context.

### Contributions to nursing

Despite its limitations, this study represents a significant advancement in nursing by applying, for the first time in Brazil, Lean Healthcare tools to critically assess bed occupancy rates in an ICU. By adopting varied management methodologies, the study promotes a strategic and evidence-based approach to improving the quality of operational processes. In addition to highlighting technical and management flaws, the proposed solutions offer concrete support for the development of standardized action plans, continuing education, and the creation of performance indicators, positioning nurses as key players in managing the quality of hygiene and patient safety. The critical and transformative role of nursing is strengthened by incorporating management tools, contributing to the creation of safer, more productive environments aligned with best healthcare practices.

## CONCLUSIONS

This study achieved its objective by analyzing the terminal hygiene process of ICU beds using Lean Healthcare tools, highlighting technical variability, waste, and critical points in the care flow, as well as guiding strategies for standardizing the procedure.

The application of Lean tools, such as VSM, Spaghetti Diagram, CRT, GUT Matrix, Prioritization Matrix, and 5W2H, allowed for the identification of relevant flaws, such as a lack of technical standardization, inadequate use of sanitizing agents, failures in the use of PPE, and deficiencies in training and monitoring process. Based on these analyses, it was possible to propose objective and feasible interventions, aligned with the real needs of the service.

Furthermore, the use of FM demonstrated that only one third of the surfaces assessed showed adequate cleanliness, reinforcing the urgency of corrective measures and continuing education.

Finally, it is observed that Lean Healthcare has proven effective in identifying waste, improving processes, and strengthening patient safety. The proposed strategies, such as the creation of checklists, illustrated materials, and targeted training, show potential for practical implementation and replication in other institutions. The standardization of the TH process, combined with continuous monitoring, represents a promising path for improving quality of care and preventing HAIs.

## Data Availability

The available research data is in the body of the article.
