# Supplementary material for: Assessment of cleaning and disinfection of Intensive Care Unit beds: quality management with Lean
Source: Rev Bras Enferm. 2026 Oct 19;79:e20250316. doi: 10.1590/0034-7167-2025-0316 (PMC13679589; doi:10.1590/0034-7167-2025-0316)
Supplement: Translation

# Translation: Avaliação da limpeza e desinfecção de leitos de terapia intensiva: gestão da qualidade com *Lean*

**DOI:** 10.1590/0034-7167-2025-0316pt

**Authors:** Joana de Oliveira Pantoja Freire, Christiany Moçali Gonzalez, Augusto da Cunha Reis, Robisom Damasceno Calado, Marluci Andrade Conceição Stipp, Graciele Oroski Paes

**Affiliations:** I Universidade Federal do Rio de Janeiro. Rio de Janeiro, RJ, Brasil; II Instituto Federal de Educação Tecnológica Celso Suckow da Fonseca. Rio de Janeiro, RJ, Brasil; III Universidade Federal Fluminense. Rio das Ostras, RJ, Brasil

**Keywords:** Desinfecção, Zeladoria Hospitalar, Indicadores de Qualidade em Assistência à, Saúde, Gestão da Qualidade Total, Unidades de Terapia intensiva.

## Abstract

**Objetivo:**

analisar, a partir da aplicação das ferramentas *Lean Healthcare*, o processo de higienização terminal em leitos de terapia intensiva e propor melhorias para sua padronização.

**Método:**

estudo transversal, realizado em hospital universitário no Rio de Janeiro entre setembro de 2023 e fevereiro de 2024, com observação de dez processos de higiene terminal executados por cinco profissionais. Utilizaram-se as ferramentas VSM, Diagrama de ARA, Matriz GUT, Matriz de Priorização e 5W2H. A análise considerou abrangência, aderência técnica, tempo de execução, completude das superfícies e presença de resíduos fluorescentes.

**Resultados:**

identificaram-se falhas no uso de equipamentos de proteção e desinfetante em 100% dos procedimentos, ausência de padrão técnico e movimentos. Apenas 70% dos processos foram completos, e 33% das superfícies estavam livres de resíduos.

**Conclusão:**

apesar da orientação institucional, não havia padronização da higiene. As ferramentas *Lean* permitiram identificar causas raízes e traçar estratégias de melhoria focadas nos reais problemas existentes.

## INTRODUÇÃO

As infecções relacionadas à assistência à saúde (IRAS) são uma importante questão de saúde pública. Elas são reconhecidas pela Organização Mundial da Saúde por sua associação a altas taxas de morbidade e mortalidade^(1,2)^. Embora algumas IRAS sejam inevitáveis, muitas podem ser prevenidas com a adoção de práticas adequadas, como a higienização das mãos e precauções padrão^(2,3)^.

A transmissão cruzada, direta ou indireta, é um fator-chave na aquisição dessas infecções, com forte influência da contaminação ambiental por microrganismos de pacientes colonizados^(3)^. Destacam-se, nesse contexto, as Unidades de Terapia Intensiva (UTIs), onde há maior gravidade clínica, uso frequente de dispositivos invasivos e, consequentemente, maior vulnerabilidade à colonização por patógenos presentes no ambiente^(2)^.

Estudos demonstram que superfícies contaminadas podem aumentar significativamente o risco de infecção, pois microrganismos hospitalares permanecem viáveis por longos períodos^(3,4)^. A higienização correta dos leitos, incluindo colchões, grades e cabeceiras, é essencial para a prevenção das IRAS, sobretudo porque superfícies frequentemente tocadas próximas ao paciente atuam como reservatórios e vetores de microrganismos multirresistentes^(2,3,5)^.

A limpeza, que antecede a desinfecção, remove sujidades visíveis e proporciona conforto ao paciente, enquanto a desinfecção visa eliminar microrganismos, garantindo maior segurança^(3)^. Quando realizada de forma sistemática e validada, a higiene hospitalar contribui significativamente para a segurança do paciente^(2,6)^.

Apesar disso, falhas nos processos de higienização são comuns, como baixa adesão à técnica, falta de monitoramento e desperdícios. Estudos apontam taxas preocupantes de conformidade em UTIs, com limpeza inadequada em áreas críticas^(7,8)^. Para enfrentar essas lacunas, é necessária a melhoria contínua da qualidade desses processos, envolvendo a enfermagem como protagonista na gestão da higienização^(9)^.

A avaliação da higienização terminal (HT) em UTIs se tornou foco de estudos voltados à segurança do cuidado. Uma abordagem promissora nesse cenário é o *Lean Healthcare*, que aplica princípios do pensamento enxuto para reduzir desperdícios e otimizar processos^(6,10)^.

Originado da indústria automobilística, o *Lean* é uma filosofia baseada na melhoria contínua, padronização e entrega de valor com o menor custo possível^(11)^. Na saúde, o *Lean Healthcare* tem se mostrado eficaz na qualificação da assistência, promovendo melhores resultados com o uso racional de recursos^(10,11)^. Hospitais no Brasil e no exterior obtiveram avanços expressivos com essa abordagem, incluindo maior eficiência operacional, redução de erros e tempo de espera, além de impacto positivo na experiência do paciente^(12-14)^.

Ainda incipiente na área da enfermagem, a aplicação do *Lean* exige mais estudos sobre seus impactos na gestão do cuidado e nos resultados clínicos. A pandemia de COVID-19 acentuou a necessidade de soluções que unam eficiência e qualidade, impulsionando o uso de ferramentas gerenciais como o *Lean* ^(11)^.

A relação entre contaminação ambiental e aquisição de IRAS é amplamente reconhecida, sobretudo pela transmissão indireta mediada por superfícies frequentemente tocadas^(4)^. Nesse contexto, a HT de leitos constitui um componente estratégico na prevenção de infecções, mas ainda apresenta variabilidade técnica e oportunidades de qualificação assistencial. O uso do *Lean Healthcare* nesse cenário possibilita analisar o leito como um microcircuito de cuidado, identificando desperdícios, mapeando fluxos operacionais e mensurando resultados com parâmetros objetivos, como o marcador fluorescente (MF)^(10-12)^. Essa abordagem confere maior precisão diagnóstica ao processo e sustenta intervenções padronizadas e reprodutíveis, reforçando a originalidade e pertinência deste estudo para a segurança do paciente e para o avanço científico e gerencial da enfermagem brasileira.

Considerando que superfícies hospitalares frequentemente tocadas estão envolvidas na transmissão de microrganismos, torna-se imperativo adotar intervenções sistemáticas para promover a melhoria contínua da limpeza e desinfecção (LD), assegurando práticas seguras e eficazes.

## OBJETIVO

Analisar, a partir da aplicação das ferramentas *Lean Healthcare*, o processo de HT em leitos de terapia intensiva e propor melhorias para a sua padronização.

## MÉTODOS

### Aspectos éticos

A pesquisa foi aprovada pelo Comitê de Ética em Pesquisa, seguindo os princípios da Resolução nº 466/2012, bem como os preceitos de autonomia, não maleficência, beneficência, justiça e equidade. Todos os participantes assinaram o Termo de Consentimento Livre e Esclarecido.

### Desenho do estudo

Trata-se de estudo observacional transversal, com abordagem quantitativa e descritiva, conduzido conforme o *checklist STrengthening the Reporting of OBservational studies in Epidemiology*. Aplicaram-se os princípios do *Lean Healthcare*, associados às ferramentas da Teoria das Restrições (TOC) (Árvore da Realidade Atual - ARA) e da Qualidade Total (Matriz GUT, 5W2H, Matriz de Priorização e Diagrama de Espaguete), para mapear e analisar o processo de HT de leitos em uma UTI.

A literatura recomenda a integração dessas ferramentas como prática eficaz na gestão e melhoria de processos assistenciais, pois favorece diagnósticos robustos e intervenções sustentadas^(15)^. O *Lean Healthcare* funciona como um arcabouço metodológico flexível, possibilitando a combinação com abordagens como *Six Sigma*, TOC e Qualidade Total^(11)^.

Complementarmente, utilizou-se o MF como instrumento objetivo para avaliar a conformidade técnica da higienização, servindo como indicador de qualidade operacional. Essa técnica mensurou se os pontos críticos de contato foram corretamente higienizados, reforçando os achados das ferramentas *Lean* ^(5,7)^.

As ferramentas foram organizadas em três fases, adaptadas ao ciclo de melhoria contínua do *Lean Healthcare*, como levantamento do processo, análise de causas e proposição de soluções sustentáveis.

### Fase I - Mapeamento, medição e identificação de falhas processuais

As ferramentas utilizadas foram Mapeamento de Fluxo de Valor (VSM), Diagrama de Espaguete e MF. O VSM permitiu visualizar o fluxo da HT, identificando falhas de sequência, retrabalho, tempos ociosos e aderência técnica. O Diagrama de Espaguete, adaptado com sequência numérica em planta baixa, representou os deslocamentos dos profissionais, evidenciando desperdícios de tempo e ergonomia. O MF, aplicado de forma cega pelo pesquisador, permitiu verificar a limpeza efetiva das superfícies previamente selecionadas^(16,17)^.

### Fase II - Análise de causas dos problemas

As ferramentas utilizadas foram ARA e Matriz GUT. A ARA da TOC possibilitou mapear logicamente os efeitos indesejados e suas causas raízes. A Matriz GUT auxiliou na priorização das falhas críticas conforme gravidade, urgência e tendência, direcionando as ações de melhoria^(18,19)^.

### Fase III - Proposição de melhorias

As ferramentas utilizadas foram Plano de ação 5W2H e Matriz Esforço x Impacto. Com base nos achados, foi estruturado um plano de ação com 5W2H, organizando de forma clara o que, como, por que e por quem as ações seriam executadas. A Matriz Esforço x Impacto auxiliou na priorização das tarefas mais viáveis e de maior retorno operacional^(19)^.

### Período e local do estudo

O estudo foi realizado entre setembro de 2023 e fevereiro de 2024 na UTI de um hospital universitário público no Rio de Janeiro. A UTI é composta por nove leitos, com média de 20 admissões mensais e permanência média de 15 dias por paciente^(20)^.

### População, amostra e critérios de inclusão e exclusão

A amostra foi censitária no período diurno, composta pelas cinco profissionais responsáveis pela higienização de leitos na UTI (quatro plantonistas 12×36 horas e uma diarista), correspondendo a 100% da equipe atuante no turno em que ocorre a maior parte das higienizações terminais. Cada auxiliar de serviços gerais (ASG) realizou dois procedimentos, totalizando dez observações.

Havia ainda quatro profissionais no plantão noturno (12×36); porém, este turno não foi incluído na coleta por apresentar menor ocorrência de altas e, consequentemente, menor número de HT durante o período do estudo.

Foram incluídos profissionais da equipe de higienização vinculados à UTI, com tempo mínimo de três meses de atuação no setor e treinamento institucional prévio e concluído para higienização em unidade crítica. A coleta contemplou exclusivamente o período diurno, no qual há maior volume de HT. Foram excluídos profissionais em férias ou afastamento durante o período da coleta, além de observações interrompidas por eventos que inviabilizassem o acompanhamento integral do procedimento.

A seleção dos leitos seguiu recomendação do *Centers for Disease Control and Prevention*, que orienta a avaliação de pelo menos 25% dos leitos em unidades críticas^(21)^. Foram definidos critérios para seleção das superfícies a serem avaliadas com MF com base em observações *in loco* e literatura científica^(1,3,4)^.

As superfícies analisadas incluíram a bomba infusora (frente e lateral), o suporte da bomba, a bomba enteral, o carrinho de curativos, o colchão, as grades (interna e externa), os controles da cama, o monitor, a régua de gases, a mesa de alimentação, o interruptor, os puxadores, a mesa de cabeceira, os botões do monitor, o dispenser de álcool e o biombo.

### Coleta de dados

Realizou-se em três fases: fase 1 - observação direta da HT, com preenchimento do VSM (tempos, interrupções, conformidades e não conformidades conformes protocolo institucional). Aplicou-se o MF (Glo Germ^®^) nas superfícies antes da higienização, sempre pelo mesmo pesquisador, garantindo sigilo operacional. O Diagrama de Espaguete foi elaborado com base na sequência das ações de limpeza sobre planta da UTI, mapeando deslocamentos e técnica empregada^(22)^; fase 2 - classificação e organização dos problemas em estrutura lógica com a ARA, seguida pela aplicação da Matriz GUT para priorização; fase 3 - estruturação do plano de ação com 5W2H e priorização pela Matriz Esforço x Impacto, conforme viabilidade e impacto das ações propostas.

### Análise dos dados

As variáveis principais foram dependentes (qualidade da HT - abrangência e aderência técnica conforme Protocolo Operacional Padrão (POP)) e independentes (tempo de execução, interrupções, uso de equipamento de proteção individual (EPI), ordem e técnica da limpeza).

Utilizaram-se as estatísticas descritivas (frequências, percentuais e médias) para análise do desempenho do processo. As ferramentas VSM e Diagrama de Espaguete foram construídas com auxílio das plataformas Miro^®^ e CorelDRAW^®^. A avaliação da fluorescência foi feita com luz UV LED, classificando a intensidade do resíduo em quatro níveis (Figura 1). O plano de ação foi modelado com base no 5W2H e na Matriz Esforço x Impacto^(7,12,22)^.

**Figura 1:** Parâmetros de análise do resíduo fluorescente sobre superfícies. Rio de Janeiro, RJ, Brasil, 2024

*Legenda: A = superfície em luz normal; B = resíduo intenso; C = resíduo moderado; D = resíduo leve.*

## RESULTADOS

### Fase I - Identificação e mensuração dos problemas

A ferramenta VSM possibilitou o mapeamento do estado atual do processo de HT do leito, a identificação das principais não conformidades que interferem diretamente em sua cadência, bem como a verificação do tempo médio de 3 horas e 10 minutos (53 minutos a 4 horas e 19 minutos) para a liberação do leito para uma nova internação (Figura 2).

**Figura 2:** Mapeamento do estado atual da higienização terminal da Unidade de Terapia Intensiva. Rio de Janeiro, RJ, Brasil, 2024

*Legenda: TP = tempo do processo; TE = tempo de espera; TI = tempo de interrupção; LT (lead time) = tempo de duração; CC = completo/correto; OM = oportunidade de melhoria.*

Os fatores que impactaram o tempo de início e a qualidade da higienização do leito foram a disponibilidade de vaga nas clínicas médicas e cirúrgicas, a urgência demandada pelo médico para admissão de novos pacientes e a sobrecarga de serviço da equipe de higienização.

Ao examinar apenas o processo de higienização, o tempo mínimo e máximo gasto do início ao fim do processo foram significativos, variando de 22 a 56 minutos. O tempo de espera e interrupção em cada etapa ocorreu principalmente para buscar materiais de limpeza.

Em todas as observações, pelo menos uma superfície não foi contemplada pelo procedimento de limpeza (mesa de cabeceira, gavetas, puxadores de gavetas, parede alta, cabeceira da cama, suporte da mesa de alimentação e parte do colchão). Por outro lado, a mesa de alimentação, as grades da cama, as áreas da parede e a mesa de cabeceira receberam fricção mecânica em quantidade superior à recomendada no POP institucional, uma vez que o processo realizado uma única vez de forma completa e correta seria eficaz.

O processo de higiene terminal do chão foi feito com uma solução composta por água, sabão, hipoclorito e desinfetante à base de quaternário de amônia e biguanida. O álcool a 70% também foi utilizado com frequência após a aplicação do desinfetante hospitalar, configurando uma segunda desinfecção das superfícies fixas.

Foram identificadas ainda inadequações na utilização de EPI, na aplicação da técnica de LD (caracterizada por movimentos que não abrangeram todas as superfícies), na prática de uso de saneantes (por meio da imersão de lenços de limpeza sujos no desinfetante, com fluxo de retorno para áreas limpas) e na instituição de rotinas de higienização diferentes para alta e para óbito.

A presença de perfurocortante em superfície sem descarte imediato e sem notificação do fato à equipe multiprofissional, assim como a falta de comunicação de perda da qualidade/integridade ou dano de equipamentos e/ou materiais, foi detectada como necessidade de melhoria do processo.

Das observações que geraram a construção do VSM, não se obteve conformidade do início ao fim, perfazendo um percentual do indicador completo e correto de 70% no processo de HT e de 80% no macroprocesso de admissão e alta da UTI.

As falhas na aplicação da técnica de higienização na unidade do paciente, identificadas no VSM, foram ilustradas no Diagrama de Espaguete adaptado, representado na Figura 3. As principais inadequações no processo foram áreas não contempladas (mesa de cabeceira e gavetas) e diversos movimentos, como vai-e-vem, zigue-zague e circular, constatando que nenhum movimento foi semelhante a outro no que diz respeito à ordem e à direção. O retrabalho da equipe ao higienizar a mesma superfície de duas a três vezes e a ausência de fluxo e de padronização do movimento foram outros aspectos observados.

**Figura 3:** Diagrama de ordem e fluxo da higienização terminal da Unidade de Terapia Intensiva. Rio de Janeiro, RJ, Brasil, 2024

Em uma das inspeções, havia um papel informativo grudado na cama e outro na parede com esparadrapo, que não foram removidos antes do processo de LD acontecer. Houve ausência de limpeza das paredes e da régua de gases por conta da altura do profissional executor, que também não verificou/buscou o cabo de suporte de limpeza.

A utilização do MF gerou 221 avaliações de 20 tipos de superfícies que compõem o ambiente do paciente, e não o ambiente de assistência em sua totalidade.

A aplicação do teste de MFs subsidiou a avaliação da qualidade da técnica de LD, o que possibilitou a identificação de falhas e ausência da LD, inclusive das superfícies visualmente higienizadas de forma completa e correta no VSM. A realização da técnica adequada de LD, representada pela retirada completa do resíduo, compôs cerca de um terço de todas as superfícies avaliadas.

As únicas áreas que obtiveram 50% ou mais de conformidade de limpeza expressos pelo resíduo ausente foram suporte da bomba infusora (75%), superfície do carrinho de curativo (54%), e grade externa da cama, bomba infusora frente, suporte do carrinho e mesa de alimentação (50%).

O biombo, os dispensadores de álcool e a bomba enteral obtiveram resultados considerados não conformes em todas as HTs observadas, uma vez que apresentaram 100% de resíduo. Biombo, dispenser de álcool fixado beira leito, painel de rede de gases, bomba enteral, interruptores e bomba infusora lateral apresentaram resíduo intenso acima de 50% na avaliação do marcador. Das 221 áreas avaliadas, 33% das superfícies altamente tocadas não sofreram nenhum tipo de LD, demonstrando que não houve qualquer fricção mecânica local.

### Fase II - Análise do problema

Para saber o motivo do processo de HT não acontecer de forma padronizada, várias ligações entre os problemas identificados foram feitas por meio de diversos “porquês”. Alguns problemas parecidos ou repetidos foram retirados, assim como outros, subjetivos e identificados a partir das falas das profissionais executoras do processo, foram inseridos, conforme a Figura 4.

**Figura 4:** Árvore da Realidade Atual dos problemas encontrados no processo de higienização terminal no leito da Unidade de Terapia Intensiva. Rio de Janeiro, RJ, Brasil, 2024

Após análise dos problemas e suas relações causais, identificaram-se cinco causas raízes da não realização da HT conforme o POP: recurso humano deficiente para distribuição de material nos setores; falta de orientação (passo a passo) para o ASG sobre o processo de LD; ausência de treinamento regular e de admissão para os ASGs; ausência de um sistema de monitoramento do processo implementado; e ausência de um sistema de incentivo e motivação profissional.

As causas raízes foram pontuadas na Matriz GUT para definir quais problemas são prioritários e merecem resolução mais urgente, de acordo com a Tabela 1.

**Tabela 1:** Matriz GUT das causas raízes da higienização terminal não padronizada. Rio de Janeiro, RJ, Brasil, 2024

Problema	Gravidade	Urgência	Tendência	G x U x T	Classificação
Falta de orientação (passo a passo) para o auxiliar de serviços gerais sobre o processo de limpeza e desinfecção	5	5	5	125	**1º**
Ausência de treinamento regular e admissional dos profissionais da higiene	5	5	5	125	**1º**
Ausência de um sistema de monitoramento do processo	5	4	5	100	**2º**
Ausência de checklist de liberação de início e término da higienização	5	5	3	75	**3º**
Canal de comunicação ineficiente da empresa com a área assistencial	3	4	4	48	4º
RH deficiente para distribuição de material nos setores	3	2	2	12	5º

Por não haver uma rotina de treinamento admissional e educação permanente dos profissionais executores da HT, percebeu-se uma falta de conhecimento no cumprimento do passo a passo do processo. Ademais, em caso de dúvidas, não há instrutivo disponível de fácil compreensão da orientação da técnica, movimento e ordem correta de LD.

### Fase III - Proposta de melhoria

O plano de ação baseado na ferramenta 5W2H abordou a implementação de oito ações de melhorias para solucionar os principais problemas relacionados ao processo de higienização hospitalar, definidos na Matriz GUT. As ações de solução incluem:

1. Facilitar o entendimento e acesso da equipe ao POP, criando uma cartilha ilustrada que descreva claramente o passo a passo da higienização (1º problema);
2. Treinar a equipe de higienização de forma dinâmica e envolvente, utilizando materiais como cartolinas e jogos, a fim de reforçar o aprendizado do POP (1º problema);
3. Implementar um treinamento inicial para novos funcionários, identificando e capacitando instrutores que serão responsáveis por acolher e treinar os novos colaboradores (1º problema);
4. Apresentar os instrutores para a equipe e valorizar sua contribuição por meio de *marketing* institucional e entrega de certificados, destacando o reconhecimento profissional (1º problema);
5. Capacitar as encarregadas sobre o processo padronizado de higienização e liderança através de treinamentos específicos que utilizam o POP e ferramentas de liderança (1º problema);
6. Implantar um sistema de monitoramento frequente do processo de HT utilizando indicadores como o MF e fornecer *feedback* contínuo à equipe (2º problema);
7. Criar o “Dia D” do profissional de higienização hospitalar junto ao *marketing*, celebrando seus resultados e promovendo o reconhecimento pelo trabalho realizado (2º problema);
8. Criar e implementar um *checklist* de liberação do leito para garantir que todas as etapas da higienização sejam verificadas antes da liberação para nova internação e criar um alerta visual de leito higienizado e não higienizado (3º problema).

Após o *brainstorming* das possíveis soluções para os problemas, com a definição do que fazer, do porquê e como fazer, onde será feito, o custo de cada ação, o período de intervenção e, por fim, quem ficará responsável pela ação de melhoria, as atividades foram priorizadas de acordo com o esforço e o impacto de cada tarefa.

Após análise da Matriz Esforço (eixo horizontal) x Impacto (eixo vertical), as ações que foram classificadas com menos exigência de esforço e impacto foram: elaborar um cronograma anual de treinamento; criar um sistema de *feedback* do processo de HT para o ASG; e criar e implementar um *checklist* de liberação de leito. A segunda prioridade de ação ficou com o quadrante de mais esforço e mais impacto, sendo elas: implementar um treinamento estruturado de admissão e permanente; criar uma cartilha ilustrada com a instrução do passo a passo da técnica de higienização; criar e implementar um indicador de avaliação de HT; e formar treinadores e multiplicadores de conhecimento do processo de HT. Por último, a ação com mais esforço e menos impacto imediato seria a criação do dia do profissional ASG como um evento institucional.

## DISCUSSÃO

Este estudo, de caráter inovador no contexto nacional, investigou as falhas e oportunidades de melhoria no processo de HT de leitos de UTI sob a perspectiva do *Lean Healthcare* ^(10-13)^. A aplicação do VSM e demais ferramentas possibilitou a visualização detalhada do processo, revelando não conformidades técnicas, interrupções, retrabalhos e desperdícios que comprometem a eficiência e a segurança assistência^(11,16)^.

Entre os principais achados, destaca-se o retrabalho como um desperdício recorrente: superfícies foram higienizadas múltiplas vezes sem ganho de efetividade, consumindo tempo e recursos desnecessariamente. Essa prática, segundo a lógica *Lean*, reflete a categoria de desperdício de movimento desnecessário, desperdício de insumo e processo inadequado^(14,16)^.

A variação técnica observada, aliada ao tempo despendido e à morfologia das superfícies, também afetou a qualidade da LD. Estudos apontam que superfícies lisas tendem a ser mais bem higienizadas que superfícies irregulares, que, apesar de mais desafiadoras, demandam maior atenção e fricção, o que nem sempre foi observado^(4,5)^.

Falhas importantes foram identificadas quanto ao uso e manipulação de saneantes, como diluições impróprias, mistura de produtos e imersão de panos contaminados na solução desinfetante comprometeram o processo. A não adoção da técnica dos dois baldes e o desconhecimento de práticas recomendadas contribuíram para a perpetuação dessas não conformidades, com risco de contaminação cruzada, como demonstrado em estudos anteriores e diluição dos desinfetantes, podendo reduzir seu efeito ou até inativar o princípio ativo^(23)^.

O uso concomitante de álcool a 70% e desinfetante hospitalar, ambos com finalidades semelhantes, configurou a categoria retrabalho. Esses achados revelam não apenas desperdícios, mas também lacunas de capacitação técnica, evidenciando a necessidade de intervenções educacionais e padronizações operacionais, conforme preconiza o princípio de Kaizen (melhoria contínua)^(11,12)^.

O tempo médio de HT observado (3 horas e 10 minutos), considerado elevado, impacta diretamente a liberação do leito para novos pacientes. Estudo internacional demonstrou que a aplicação de VSM e Kaizen em ambiente hospitalar pode reduzir significativamente esse tempo, com ganhos em eficiência e satisfação do paciente^(10,16)^.

Apesar da rotina estabelecida para descarte de material perfurocortante, foram observadas falhas pontuais, como o não descarte imediato e a ausência de notificação interna. Tal fragilidade expõe os profissionais a riscos ocupacionais, indicando necessidade de reforço nos protocolos e treinamentos^(3,5)^.

A integridade dos materiais também interferiu na efetividade da limpeza. Superfícies danificadas, como colchões rasgados, inviabilizam a desinfecção adequada e favorecem a formação de biofilmes. A adoção de inspeções visuais, auditorias e testes de impermeabilidade se mostra fundamental para a segurança do paciente^(4,23)^.

A observação direta revelou a ausência de um padrão técnico nas etapas da HT. O Diagrama de Espaguete adaptado evidenciou variações na sequência das ações e deslocamentos, reforçando a necessidade de padronização. O uso do MF complementou essa análise, revelando superfícies aparentemente limpas, mas que, sob luz UV, apresentaram resíduos intensos, sugerindo falhas na técnica de limpeza^(8,16,21)^.

Quando os resíduos ausentes (indicadores de limpeza completa e correta) foram isolados, constatou-se que apenas cerca de 33% das superfícies atendiam aos critérios. Isso significa que, a cada dez leitos liberados, apenas três estariam adequadamente higienizados para receber um novo paciente^(5,6)^.

Estudos demonstram que o uso do MF associado ao *feedback* imediato pode melhorar a adesão às práticas corretas de limpeza. No presente estudo, observou-se ausência de retorno sistemático sobre o desempenho dos profissionais, sendo o *feedback*, quando existente, predominantemente negativo. O retorno positivo e educativo tem potencial de atuar como incentivo e valorização do trabalho da equipe de limpeza^(5,21,24)^.

A análise utilizando a ARA evidenciou que as principais causas das falhas foram a falta de orientação formal sobre o passo a passo da HT, a ausência de treinamento sistemático e a inexistência de materiais de consulta para os profissionais. Isso reforça a importância de desenvolver ações estruturadas de capacitação, promover o pensamento crítico e implementar uma cultura de melhoria contínua^(11,17)^.

A construção de planos de ação com base no 5W2H e a priorização das tarefas pela Matriz Esforço x Impacto permitiram delinear propostas viáveis e de alto impacto. Entre elas, destaca-se a criação de um *checklist* para início e término da HT, alinhado ao POP e validado pelas observações do VSM, visando garantir a segurança do processo e facilitar sua execução^(12)^.

O pensamento *Lean* oferece uma abordagem estratégica para revisão e aprimoramento de processos de saúde. Ao eliminar desperdícios e promover a padronização, contribui para a segurança do paciente, a eficiência operacional e a sustentabilidade dos serviços. Apesar de sua aplicação ainda ser limitada na área da higiene hospitalar, os resultados deste estudo reforçam seu potencial transformador^(10,13)^.

### Limitações do estudo

Apesar da consistência metodológica, este estudo apresenta limitações que devem ser reconhecidas. A coleta ocorreu em uma única UTI, o que restringe a extrapolação dos achados para outros cenários com dinâmicas assistenciais, estruturas físicas e culturas organizacionais distintas. A amostra, embora reduzida, foi censitária no período diurno, contemplando a totalidade das profissionais responsáveis pela higienização dos leitos, o que justifica o número amostral e mitiga vieses de seleção. Ainda assim, a não inclusão do plantão noturno pode ter limitado a variabilidade comportamental analisada. Outra limitação refere-se à ausência de avaliação microbiológica das superfícies, que poderia complementar e aprofundar os achados obtidos com MF e observação direta. No entanto, os instrumentos empregados demonstraram sensibilidade adequada para identificar falhas técnicas e orientar melhorias no processo de higienização, sugerindo o potencial de replicação do modelo de análise em outras unidades e instituições, mediante adaptação ao contexto local.

### Contribuições para a área da enfermagem

Apesar das limitações, este estudo representa um avanço significativo no campo da enfermagem ao aplicar, de forma inédita no Brasil, ferramentas do *Lean Healthcare* para avaliar criticamente a HT de leitos em uma UTI. Ao adotar metodologias de gestão variadas, o estudo promove uma abordagem estratégica e baseada em evidências para melhoria da qualidade dos processos operacionais. Além de apontar falhas técnicas e de gestão, as soluções propostas oferecem subsídios concretos para o desenvolvimento de planos de ação padronizados, formação continuada e criação de indicadores de desempenho, inserindo o enfermeiro como protagonista na gestão da qualidade da higienização e segurança do paciente. A atuação crítica e transformadora da enfermagem é fortalecida ao incorporar ferramentas gerenciais, contribuindo para a construção de ambientes mais seguros, produtivos e alinhados às boas práticas assistenciais.

## CONCLUSÕES

O presente estudo atingiu seu objetivo ao analisar o processo de higienização terminal de leitos de terapia intensiva a partir das ferramentas *Lean Healthcare*, evidenciando variabilidade técnica, desperdícios e pontos críticos do fluxo assistencial, além de direcionar estratégias para a padronização do procedimento.

A aplicação das ferramentas *Lean*, como o VSM, o Diagrama de Espaguete, a ARA, a Matriz GUT, a Matriz de Priorização e o 5W2H, permitiu identificar falhas relevantes, como ausência de padronização técnica, uso inadequado de saneantes, falhas no uso de EPIs e deficiência em capacitação e monitoramento do processo. Com base nessas análises, foi possível propor intervenções objetivas e factíveis, alinhadas às necessidades reais do serviço.

Além disso, o uso do MF demonstrou que apenas um terço das superfícies avaliadas apresentou limpeza adequada, reforçando a urgência de medidas corretivas e educação permanente.

Por fim, observa-se que o *Lean Healthcare* se mostrou eficaz na identificação de desperdícios, qualificação do processo e fortalecimento da segurança do paciente. As estratégias propostas, como a criação de *checklists*, materiais ilustrados e treinamentos direcionados, apresentam potencial de implementação prática e replicação em outras instituições. A padronização do processo de HT, aliada ao monitoramento contínuo, representa um caminho promissor para a melhoria da qualidade da assistência e prevenção de IRAS.

## Data Availability

Os dados de pesquisa disponíveis estão no corpo do artigo.
